# Community-Based Intelligent Blood Glucose Management for Older Adults With Type 2 Diabetes Based on the Health Belief Model: Randomized Controlled Trial

**DOI:** 10.2196/60227

**Published:** 2025-06-19

**Authors:** Anqi Zhang, Jinsong Wang, Xiaojuan Wan, Ziyi Zhang, Shuhan Zhao, Shuo Bai, Yamin Miao, Shuang Yang, Xue Jiang

**Affiliations:** 1 The Affiliated Hospital of Yangzhou University, Yangzhou University, No. 368, Hanjiang Middle Road, Yangzhou City, Yangzhou, 225009, China, 86 15952771632; 2 School of Nursing and Public Health, Yangzhou University, Yangzhou, China; 3 Mufu Mountain Community Health Service Center,Nanjing Gulou District Health Commission, Nan jing, China

**Keywords:** older adults, Type 2 diabetes, telemedicine, health belief model, glycosylated hemoglobin, blood glucose, glucose, glucose management, community, community based, home intelligent, home telemedicine, health education, community dwelling, randomized controlled trial

## Abstract

**Background:**

The number of older patients with type 2 diabetes (T2D) is increasing, and effective self-management is crucial for controlling disease progression and its complications.

**Objective:**

We designed a home telemedicine intervention that combines telemedicine with health education based on the Health Belief Model (HBM). This study evaluated its effectiveness on self-management in older patients with T2D.

**Methods:**

Between March and April 2022, we recruited 198 community-dwelling patients with T2D aged 65 years and older. Patients were randomly assigned to either a control group, which received a conventional diabetes management program, or an intervention group, which received a home telemedicine intervention with a health education program based on the HBM. The intervention lasted 6 months. The primary outcome measured was glycosylated hemoglobin (HbA_1c_); secondary outcomes included diabetes self-management capacity, self-efficacy, and health beliefs. We collected outcome metrics at baseline, 3 months, and 6 months. Generalized estimating equations were used to compare changes in outcomes.

**Results:**

A total of 96.5% (191/198) of patients completed the study. From baseline to 6 months, HbA_1c_ decreased by mean −0.99% (95% CI −1.60% to −0.60%) in the intervention group and mean −0.42% (95% CI −0.90% to 0.90%) in the control group. The intervention group experienced a significantly greater reduction of 0.42% compared to the control group (95% CI 0.12%-0.73%). Furthermore, compared to the control group, the intervention group showed significant improvements in diabetes self-management skills (mean 5.88, 95% CI 4.98-6.79), self-efficacy (mean 9.40, 95% CI 8.15-10.66), and health beliefs (mean 19.54, 95% CI 17.71-21.36) at both 3 and 6 months.

**Conclusions:**

Home telemedicine interventions incorporating health education based on the HBM can provide significant benefits for community-dwelling older patients with T2D, potentially offering new avenues for chronic disease prevention and management. However, future large-scale studies are required to further assess their effectiveness and feasibility.

## Introduction

The global diabetes population has quadrupled in the last 30 years, making diabetes the ninth leading cause of death [1]. In China, the prevalence of diabetes has rapidly increased from less than 1% in 1980 to 11.9% in 2022 [2], ranking it the second highest in the world [3]. The incidence of diabetes mellitus in China is positively correlated with aging, with a prevalence of 11.5% among the middle-aged population (45‐59 y old) and over 20% among the older individuals (≥65 y old) [4]. Older adults with type 2 diabetes (T2D) are at higher risk of comorbidities and complications. Approximately one-fifth of older patients with T2D develop serious complications [5], such as cardiovascular disease, psychiatric disorders, and physical dysfunction [1]. This imposes a significant burden on both society and the individual [6]. Consequently, providing adequate health care services to enable effective diabetes management has become critical.

Emerging telemedicine practices may address these needs by facilitating the delivery of health care services and health education to patients across geographies via the internet [7]. Particularly for chronic diseases like T2D, which require repeated consultations with physicians, telemedicine offers a viable alternative for patients seeking medical guidance [8]. Telemedicine typically supports four functions: (1) health promotion and knowledge dissemination, (2) timely medical and nursing support, (3) remote disease monitoring, and (4) decision support systems [9]. Given the limitations of health care service systems and the use of 5G networks, remote medical interventions represent a new opportunity to improve chronic disease management for patients with T2D [10]. Since the majority of patients with T2D are treated in community hospitals, telemedicine can help patients benefit from long-term monitoring and regular self-management [11].

Telemedicine interventions guided by health theories may yield better results [12]. The Health Belief Model (HBM) is an important theoretical framework that uses social psychological approaches to explain and predict changes in health-related behaviors, and it is widely used in health behavior research [13]. Studies show that applying the HBM to diabetes management can enhance knowledge [14], improve self-care behaviors [15], promote healthy lifestyles [14], and encourage active participation in disease management [16]. HBM has been used as a theoretical foundation for developing mobile health or telemedicine interventions [17], and many of its constructs have been demonstrated to effectively predict a range of health behaviors in meta-analyses [18]. The model is designed to help patients understand the severity of their disease, provide strategies for management, increase self-efficacy, enhance health behaviors, and achieve better health outcomes [19]. Therefore, given the importance of behavior change in telemedicine interventions, HBM may be a particularly relevant theory to enhance the impact of such interventions on self-management behaviors in older adults with T2D.

However, the effectiveness of such telemedicine interventions in community-dwelling older adults with T2D has not yet been established. Studies have indicated that telemedicine users are generally younger, while older or less-educated individuals have lower usage rates, placing them at a relative disadvantage [20]. Moreover, mismatches between app design and user capabilities, along with the poor quality of diabetes apps, pose significant challenges to telemedicine implementation [21]. To date, there is a notable lack of directly applicable clinical trial data on telemedicine interventions for community-dwelling older adults with T2D.

To provide clinical evidence to inform treatment recommendations for community-dwelling older adults, we conducted a randomized controlled trial of a telemedicine intervention among this group with T2D. We evaluated the effectiveness of a comprehensive, personalized education, and behavioral intervention program, based on the HBM and telemedicine, in improving home self-management for these older adults.

## Methods

### Recruitment

From March 2022 to April 2022, we recruited participants with T2D from a community hospital in Yangzhou City.

The inclusion criteria were as follows: (1) age 65 years and older; (2) type 2 diabetes mellitus: diabetes was diagnosed based on self-reports with verification (current treatment, medical records, confirmation from health care providers, fasting plasma glucose of ≥126 mg/dL, or symptoms of hyperglycemia with a plasma glucose level of ≥200 mg/dL); (3) informed consent and voluntary participation in the study; and (4) capability for self-care, no communication barriers, and no cognitive impairment.

The exclusion criteria were as follows: (1) serious physical illnesses (eg, malignant tumors; severe cardiac, hepatic, or renal insufficiency; and cerebrovascular accidents), (2) serious diabetes mellitus–related comorbidities, and (3) current participation in other clinical trials or plans to participate in other clinical trials.

Researchers screened eligible participants through electronic health records within the community hospital health care system and contacted them by phone. Participants who agreed to participate in the study were invited to the community hospital, where researchers met them face to face to discuss the details of the intervention program again, following informed consent. Laboratory testing and questionnaire collection were completed for eligible participants. A total of 198 participants were recruited. At 3 and 6 months of intervention, nurses reminded all participants to visit the community hospital for laboratory examinations and to complete the corresponding questionnaire collection.

### Randomization and Masking

Using Microsoft Excel to generate random numbers, eligible study participants were randomly divided into an intervention group and a control group, each comprising 99 individuals, thus maintaining a 1:1 ratio. Recruitment personnel, data collectors, and data analysts were kept unaware of the intervention allocation. Recruitment, data collection, and data analysis were conducted by the respective research assistants, researchers, and statisticians. The physician conducting the lab tests was also unaware of the group assignments. Due to the nature of the intervention, the health care provider delivering the intervention was not blinded.

#### Control Group

Routine community-based diabetes management was implemented, including a free health consultation yearly and random blood glucose testing every 3 months. Participants also received diabetes health management handbooks.

#### Intervention Group

#### Overview

In addition to routine community diabetes management and receiving diabetes health management handbooks, participants in this group were managed by a team comprising 8 doctors, 2 pharmacists, 20 nurses, and 1 information engineer. The interventions included (1) telemedicine smart devices and their manuals for information collection and enhancing doctor-patient communication, (2) provision of a glucose meter for self-monitoring of blood glucose, (3) telemedicine guidance from a multidisciplinary team, and (4) personalized diet and exercise plans.

##### Home Telemedicine Interventions

The “Xiaodu Smart Screen X8 (Baidu)” telemedicine smart device connects via Bluetooth to a glucometer. Health information collected from the smart device, including blood sugar levels, exercise, diet, weight, and blood pressure, is automatically transmitted over wireless networks to a centralized website. This setup allows a multidisciplinary team to easily access and analyze the data, motivating participants to maintain self-management through ongoing personal monitoring and intervention.

Participants self-measured their blood sugar at least twice a week during the study. They chose the timing of each measurement, either fasting or 2 hours post meal. If a patient’s blood glucose value deviates from the normal range, the system triggers an automatic alert on the physician’s side. Within 3 minutes of receiving the alert, the physician contacts the patient by phone to assess whether medical attention is needed. The doctor also determines the cause of the abnormal values and provides appropriate medical guidance. If a patient’s blood glucose value has not been uploaded for more than a week, a nurse calls the patient to remind them.

Pharmacists provide guidance to participants on improving blood glucose levels and offer medication recommendations based on individual glucose control. Participants in the intervention group used a smart device to voice-record their daily medication intake, and pharmacists received these records along with blood glucose data for each patient via the physician. Based on data analysis, the health care team sent a monthly medication management program to participants in the intervention group.

Participants could also input health records, including exercise, diet, weight, and blood pressure, through voice or manual entry on their smart devices. The device then generated personalized diet and exercise regimens based on their conditions. The multidisciplinary team accessed the website every 2 weeks to review the participants’ diet and exercise plans and sent nutritional education information through the smart devices.

In addition, the smart devices featured a one-click medical alert and enabled bidirectional communication between participants and the health care team via a chat function.

### Health Guidance by the Multidisciplinary Team

Upon their initial inclusion in the study, all participants received a diabetes self-management handbook based on the HBM. The multidisciplinary team, consisting of physicians, nurses, and pharmacists, had undergone specialized training in diabetes management intervention based on the HBM, equipping them with expertise in diabetes management and health coaching. Each group of 25 intervention participants was assigned to a team that provided face-to-face group education sessions every 2 weeks for 3 consecutive months (40‐50 min each). These sessions took place in the training room of the community hospital. The intervention group was divided into 4 groups, each comprising approximately 25 individuals. Each group attended educational sessions scheduled for the same week. The educational content was structured around the dimensions of the HBM and covered topics, such as the definition, symptoms, and risk factors of T2D (perceived susceptibility); benefits of using smart devices for diabetes management (perceived benefits); impact of T2D on quality of life and physical health (perceived severity); diabetes self-management through smart devices (cues to action); discussion of the patient’s experience in self-management and use of smart devices, providing solutions to reduce and remove barriers (perceived barriers); and mobilizing family support, showcasing role models, and increasing self-efficacy (self-efficacy). Each session featured a lecture and discussion on the week’s topic, incorporating photos, videos, presentations, and other relevant materials.

### Outcomes and Measures

Participants’ baseline data were collected using a researcher-designed individual health information form, which included age, sex, living arrangements, educational level, marital status, medical insurance coverage, employment status, monthly household income, duration of diabetes, treatment methods, complications, comorbidities, and other demographic information.

#### Primary Outcome

The primary outcome was the level of glycated hemoglobin (HbA_1c_), assessed at baseline, 3 months, and 6 months. A nurse collected a fasting venous blood sample through venipuncture. The samples were processed by the diagnostic testing laboratory at the community hospital and measured using high-performance liquid chromatography on a Tosoh Automatic Glycohemoglobin Analyzer HLC-723G8.

#### Secondary Outcomes

##### The Summary of Diabetes Self-Care Activities Measure

The summary of diabetes self-care activities measure (SDSCA) was used to measure the frequency of self-management practices in individuals with diabetes over the past 7 days. This scale, designed by Toobert et al [22], includes 11 items divided into six dimensions: general diet, special diet, blood glucose monitoring, physical activity, foot care, and smoking. It uses a 7-point Likert scoring system, where the score for each dimension is the mean score of the items within that dimension. A higher total score indicates better self-management behavior.

##### The Self-Efficacy for Diabetes

The study used the Chinese version of the self-efficacy for diabetes (SED), originally developed by Lorig and translated and revised by Wei [23]. This scale assesses participants’ self-efficacy in managing their glycemia, diet, exercise, and disease monitoring. It includes 9 items scored on a 5-point Likert scale, with higher scores indicating greater self-efficacy.

##### Health Beliefs Questionnaire

The study used a health belief questionnaire designed by Chen [24], based a previously proposed HBM framework. Tailored for individuals with diabetes, this questionnaire measures their health beliefs across 20 items, distributed among 5 dimensions: perceived benefits, perceived severity, perceived susceptibility, perceived barriers, and cues to action. Scoring used a 5-point Likert scale, where higher scores indicate stronger health beliefs.

### Sample Size Calculation

This study used HbA_1c_ values as the primary outcome indicator. We set the reference values based on previous literature [25]. The mean HbA_1c_ in the treatment group was 9% (SD 1.9%). In the control group, the mean HbA_1c_ was 10% (SD 2.7%). We set a 2-sided α of 0.05 with 80% power. Using G*Power 3.1 software (Heinrich-Heine-Universität Düsseldorf), the sample size for the treatment group (N1) was calculated to be 87 cases, and for the control group (N2), it was 87 cases. Accounting for a potential dropout and refusal rate of 10%, we determined that a minimum of 96 participants in each of the intervention and control groups was needed, totaling a minimum of 192 participants.

### Statistical Analysis

Statistical analysis in this study was conducted using SPSS 27.0 (IBM Corp), R (version 4.0.3, R Core Team), and GraphPad Prism (version 9.4.1). A *P* value <.05 was considered statistically significant.

Descriptive statistics were used to describe the demographic distribution and scores for the SDSCA, SED, and HBM. Variables with normal distribution were expressed as mean (SD), categorical variables were expressed as frequencies (percentages), and non-normally distributed variables were expressed as medians (quartiles). Continuous values were compared to baseline data using the independent samples *t* test or Mann-Whitney *U* test.

The study used an intention-to-treat analysis to analyze the data. With no pattern in the missing data, the 10% missing values were assumed to be missing at random. The predicted values of the missing data were estimated using Markov Chain Monte Carlo multiple imputation to obtain a complete dataset. For the primary outcome (6-month change in HbA_1c_), linear mixed-effects models were used with HbA_1c_ as the dependent variable. This model included a random intercept with an unstructured covariance matrix, indicator variables for time and study group, and group-by-time interaction terms. The same approach was used for the secondary outcomes.

### Ethical Considerations

The study protocol adhered to the Declaration of Helsinki and received approval from the Ethics Committee of the School of Nursing and Public Health at Yangzhou University (approval number YZUHL2022001). The trial has been developed and reported in agreement with the CONSORT (Consolidated Standards for Reporting Clinical Trials) checklist (Checklist 1). Written informed consent was obtained from all participants.

To ensure privacy and security during the use of the device, each participant must log in to the device with a unique account and password. After data collection, it is anonymized and stored in the cloud server, strictly following advanced data protection regulations. At the same time, the system will perform encrypted backup operations as needed to ensure that data and programs are protected.

## Results

### Participant Characteristics

**Figure 1.**
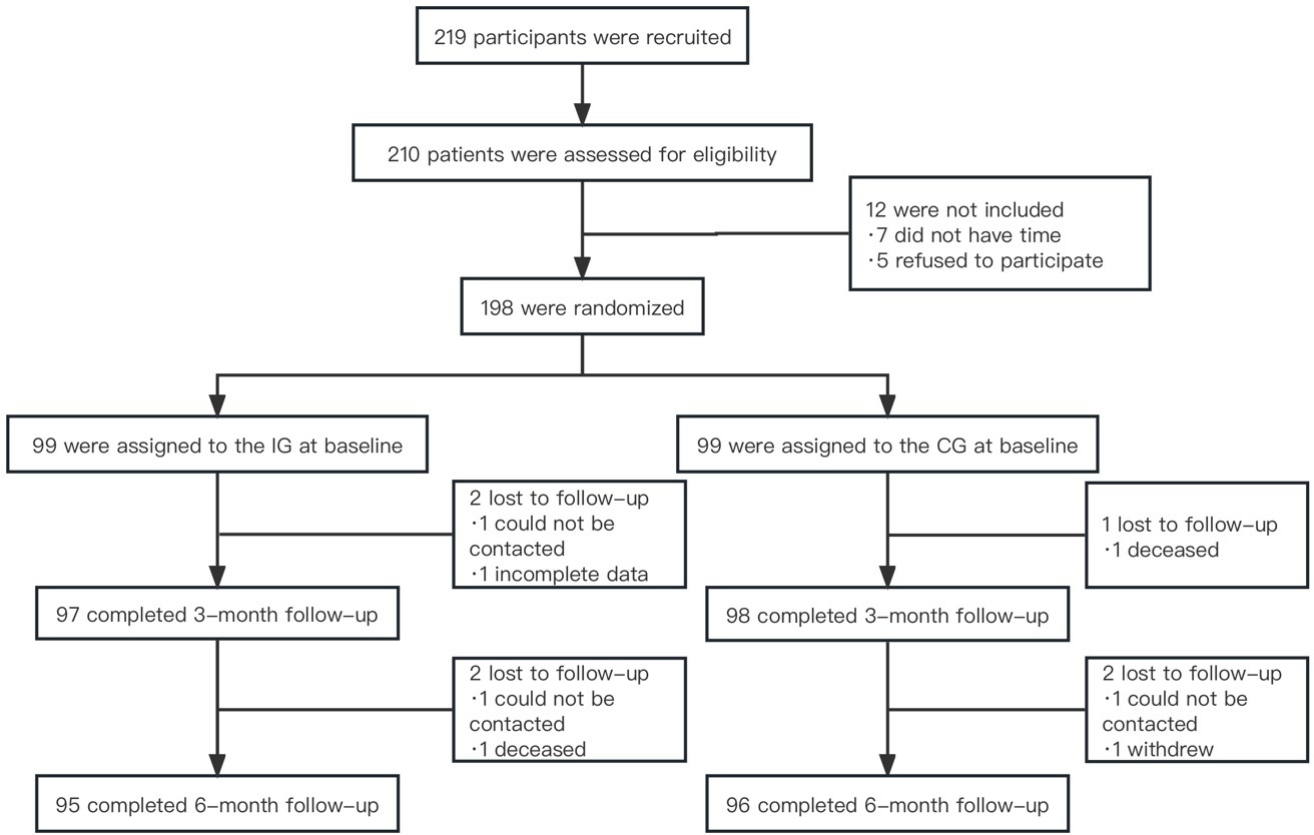
Flowchart of the study. IG: Intervention group; CG: Control group

A total of 219 potential participants were assessed for eligibility in this study. Out of the 210 who were eligible, 12 were excluded due to scheduling conflicts and a lack of interest. Consequently, 198 individuals agreed to participate and were randomly assigned to either the intervention group (n=99) or the control group (n=99) after providing informed consent. A total of 198 out of 191 (96.5%) participants completed the follow-up assessments (Figure 1). The ages of the 198 participants ranged from 65 to 87 years. The duration of diabetes varied from 1 to 41 years, with a mean HbA_1c_ level of 7.05% (SD 2.41%, range 6.20%-8.25%). In addition, 76.8% (198/152) of the participants were taking oral hypoglycemic drugs to control their blood glucose, 22.7% (198/45) had complications, and 29.3% (198/58) had 2 or more comorbidities. There were no significant differences in baseline characteristics between the 2 groups (*P*>.05), as shown in Table 1.

**Table 1.** Demographic and clinical characteristics of the participants (N=198).

Variables	Intervention group (n=99)	Control group (n=99)	*t* test^a^/Mann-Whitney *χ*^2b^ (degrees of freedom for *t* test=196)	*P* value
Age (years), mean (SD)	72.82 (4.85)	74.11 (5.49)	–1.621^a^	.11
Disease duration (years), mean (SD)	12.67 (7.96)	13.61 (9.83)	–0.266^a^	.79
Sex, n (%)			–1.703^b^	.09
Male	53 (53.54)	44 (44.44)	—^c^	—
Female	46 (46.46)	55 (55.56)	—	—
Living arrangements, n (%)			–0.097^b^	.92
Living alone	7 (7.07)	8 (8.08)	—	—
Living with spouse	30 (30.30)	31 (31.31)	—	
Living with family	62 (62.62)	60 (60.61)	—	—
Educational level, n (%)			–1.871^b^	.06
Elementary school or below	32 (32.32)	47 (47.47)	—	—
Junior high school	38 (38.38)	29 (29.29)	—	—
High school	24 (24.24)	18 (18.18)	—	—
University and above	5 (5.06)	5 (5.06)	—	—
Marital status, n (%)			–0.420^b^	.68
Married	85 (85.85)	87 (87.88)	—	—
Divorced or widowed	14 (14.15)	12 (12.12)	—	—
Medical insurance coverage, n (%)			–1.219^b^	.22
Employees health insurance	82 (82.82)	76 (76.77)	—	—
Residential health insurance	10 (10.10)	8 (8.08)	—	—
Commercial health insurance	7 (7.08)	15 (15.15)	—	—
Employment status, n (%)			–1.790^b^	.07
Employed	1 (1.06)	4 (4.12)	—	—
Unemployed	4 (4.04)	8 (8.08)	—	—
Retired	94 (94.95)	87 (87.88)	—	—
Monthly household income (RMB^d^), n (%)			–0.064^b^	.95
<3000	15 (15.15)	21 (21.21)	—	—
3000-5000	33 (33.33)	24 (24.24)	—	—
5000-10000	45 (45.45)	46 (46.46)	—	—
>10000	6 (6.07)	8 (8.09)	—	—
Treatment method, n (%)			–1.330^b^	.18
None	0	10 (10.10)	—	—
Oral medication	81 (81.82)	71 (71.72)	—	—
Insulin	6 (6.06)	5 (5.05)	—	—
Oral medication+ insulin	12 (12.12)	13 (13.13)	—	—
Comorbidities, n (%)			–1.583^b^	.11
No	11 (11.11)	25 (25.25)	—	—
One	58 (58.59)	46 (46.47)	—	—
Two	26 (26.26)	25 (25.25)	—	—
Three	4 (4.04)	3 (3.03)	—	—
Complications, n (%)			–1.867^b^	.06
No	71 (71.72)	82 (82.83)	—	—
One	26 (26.26)	16 (16.16)	—	—
Two	2 (2.02)	1 (1.01)	—	—
HbA_1c_ ^e^, mean (95% CI)	7.3 (6.30-8.50)	6.90 (6.10-8.90)	–0.514^a^	.61
SDSCA ^f^, mean (SD)	16.86 (5.02)	17.18 (4.83)	–1.321^a^	.19
SED ^g^, mean (SD)	25.37 (5.01)	26.28 (3.34)	–1.197^a^	.23
HBM ^h^, mean (SD)	69.53 (7.56)	71.59 (4.71)	–1.110^a^	.27

a *t* test.

b Chi-square values derived from the Mann-Whitney *U* test.

c Not applicable.

d RMB: Renminbi; RMB 1=US $0.14.

e HbA_1c_: glycated hemoglobin.

f SDSCA: The Summary of Diabetes Self-Care Activities Measure.

g SED: The Self-efficacy for Diabetes.

h HBM: health belief model.

### Primary Outcome

Participants’ HbA_1c_ levels decreased after 3 months of intervention (*P*=.02), with a more significant improvement noted at 6 months (*P<.*001). In the intervention group, participants’ HbA_1c_ levels showed better control during follow-up (*P*<.001). The decrease in HbA_1c_ levels at 6 months in the control group was not statistically significant (*P*=.06). The interaction between group and time was not statistically significant (*P*=.51), as shown in Table 2. In the intervention group, HbA_1c_ levels decreased from a baseline of 7.87% to 7.28% at 3 months and further to 6.88% at 6 months. In the control group, HbA_1c_ levels decreased from a baseline of 7.72% to 7.30% at 6 months. These changes are illustrated in Figure S1 in Multimedia Appendix 1.

**Table 2.** The impact of home telemedicine interventions based on the Health Belief Model on glycated hemoglobin levels in older adults with type 2 diabetes.

Outcome measurement		𝛽^a^	SE (95% CI)			*P* value
HbA_1c_ (%)^b^						
Group^c^ effect		8.02	0.404 (7.228 to 8.812)			<.001
Time^d^ 2		−1.04	0.451 (−1.924 to −0.156)			.02
Time^d^ 3		−1.56	0.451 (−2.444 to −0.676)			<.001
Group time^e^ 2		0.45	0.285 (−0.109 to 1.009)			.11
Group time^e^ 3		0.57	0.285 (0.011 to 1.129)			.05

a 𝛽: regression coefficient.

b HbA_1c_: glycated hemoglobin.

cc group effect: difference between groups at baseline.

d time 2, time 3: time effects at 3 and 6 months compared to baseline.

e group*time effect: difference in change at 3 and 6 months postintervention compared to baseline.

### Secondary Outcomes

At 3 months of intervention, mean SDSCA scores increased from baseline levels in both the intervention (17.13, SD 24.50 vs 19.59, SD 21.54) and control groups (16.97, SD 22.52 vs 18.43, SD 17.58). At 6 months, mean SDSCA scores were higher than baseline levels in the intervention group (17.13, SD 24.50 vs 22.93, SD 14.59; *P*<.001), whereas mean SDSCA scores were lower than baseline levels in the control group (16.97, SD 22.52 vs 16.93, SD 22.49). The difference in scores between the 2 groups was statistically significant (*P<*.001).

Mean SED scores also improved from baseline in both groups at 3 months: the intervention (25.38, SD 25.38 vs 29.51, SD 28.25) and control groups (26.18, SD 11.16 vs 26.44, SD 7.55). At 6 months, mean SED scores were higher than baseline levels in the intervention group (25.38, SD 25.38 vs 34.99, SD 17.95; *P<*.001), while mean SED scores were slightly higher than baseline levels in the control group (26.18, SD 11.16 vs 26.24, SD 8.75). There was a statistically significant difference between the 2 groups (*P<*.001) at both 3 and 6 months.

At 3 months, HBM scores were higher than baseline in both the intervention group (69.91, SD 56.44 vs 77.89, SD 29.95) and the control group (1.55, SD 22.12 vs 73.35, SD 19.73). At 6 months, HBM scores were significantly higher than baseline levels in both the intervention group (89.65, SD 18.44) and the control group (77.04, SD 23.05), and the intervention group showed a greater improvement in HBM scores. There was a statistically significant difference between the 2 groups (*P<*.001) at both 3 and 6 months, as detailed in Table 3 and Figure S2 in Multimedia Appendix 1.

**Table 3.** Changes in secondary outcomes during the home telemedicine intervention compared to baseline.

Variables and time		Intervention group, mean (95% CI)	Control group, mean (95% CI)	Difference between groups, mean (95% CI)
SDSCA ^a^-compared to Baseline				
Change in 3 months		2.58 (1.27 to 3.88)	1.34 (0.17 to 2.51)^b^	–0.97 (–2.19 to 0.26)
Change in 6 months		5.88 (4.98 to 6.79)^c^	–0.11 (–1.34 to 1.14)	–5.72 (–6.89 to –4.55)^c^
SED ^d^-compared to baseline				
Change in 3 months		3.98 (2.72 to 5.24)^c^	0.18 (–0.23 to 0.60)	–3.15 (–4.22 to –2.07)^c^
Change in 6 months		9.40 (8.15 to 10.66)^c^	0.05 (–0.49 to 0.59)	−8.70 (−9.68 to −7.66)^c^
HBM-compared to Baseline				
Change in 3 months		8.42 (6.54 to 10.30)^c^	1.71 (1.02 to 2.39)^c^	–4.89 (–6.21 to –3.57)^c^
Change in 6 months		19.54 (17.71 to 21.36)^c^	5.52 (4.43 to 6.60)^c^	–12.19 (–13.57 to –10.81)^c^

a SDSCA: The Summary of Diabetes Self-Care Activities Measure.

b *P<*.05.

c *P<*.001.

d SED: The Self-efficacy for Diabetes.

e HBM: Health Belief Model.

### Adverse Events

No serious adverse clinical events were reported from enrollment to the 6-month follow-up. However, some operational issues with the devices, such as Bluetooth pairing problems between blood glucose meters and smart devices, were reported as inconvenient by participants. Indeed, it has been demonstrated that such issues can lead to lower satisfaction and reduced use of remote technology [26]. Before the start of the study, all participants were informed about the potential for these problems and the availability of telephone assistance.

## Discussion

### Principal Findings

In this study, we tested a home telemedicine intervention and health education program based on the HBM among community-recruited older patients with T2D. Screening, recruitment, and participation outcomes indicated that, although the number of participants was small, the vast majority were willing to participate and complete the program. While adherence was high in both groups, adherence to self-management varied significantly. Overall, this program has shown potential in helping older patients with T2D achieve safe and effective home diabetes self-management under the supervision and guidance of health care providers.

Longitudinal analyses demonstrated significant improvements in HbA_1c_ levels at 3 and 6 months, as well as significant enhancements in diabetes self-management, self-efficacy, and health belief scores in the intervention group. In contrast, in the control group, only health beliefs showed significant improvement at 6 months. HbA_1c_ levels were significantly lower following the intelligent blood glucose management in the intervention group compared to the control group. This study underscores the effectiveness of intelligent blood glucose management as a means of delivering behavioral health interventions to older adults with T2D [27]. At 3 months, HbA_1c_ levels in the intervention group decreased significantly by 0.59% from baseline, compared to 0.13% in the control group; however, this difference was not statistically significant. At 6 months, HbA_1c_ levels in the intervention group further decreased by 0.40% compared to 0.29% in the control group, indicating sustained improvement. This finding is consistent with research by Sun et al [28], suggesting that older adults may require time to familiarize themselves with telemedicine systems. Furthermore, after training and remote guidance from the health care team, participants were better able to use smart devices independently [29]. In this study, glycemic management improved in both groups, which may be attributed to several factors. First, the Hawthorne effect [30] could explain improvements, as control participants also had frequent contact with researchers and received additional attention, including regular tests of their glycosylated hemoglobin every 3 months. Second, all participants received a diabetes self-management handbook and specialized care from health care professionals, potentially allowing for more intensive glucose management than usual [31]. However, sustained and effective long-term effects are challenging without ongoing telemedicine oversight and support [32]. Therefore, long-term follow-up of older adults with T2D is crucial.

The significant and long-lasting improvement in self-management in the intervention group, compared to the control group, suggests that our intervention may promote improved self-management behaviors, aligning with findings from previous studies [33]. However, one study indicated that the difference between groups using intelligent blood glucose management alone was not significant, possibly due to older adults’ lack of experience with and incomplete knowledge of smart devices [34]. After incorporating group health education based on the HBM, participants in the intervention group significantly improved their self-management skills. This underscores the vital role that face-to-face diabetes health education plays in helping older adults overcome the challenges of using smart devices and enhancing their self-management. A systematic evaluation noted that an essential component of diabetes self-management is the active participation of the patient in their care [8]. One effective method is through health education, which facilitates the learning process of self-management. Ahmad et al [35] found that older adults with T2D may require regular face-to-face counseling to address the challenges they encounter during telemedicine interventions. Providing support and education through a dedicated community-based diabetes health care team can help participants achieve optimal self-management [31]. Therefore, combining telemedicine with face-to-face health education appears to be a promising approach for enhancing diabetes self-management.

In this study, the health belief scores of participants in the intervention group were higher than those in the control group. The longer the intervention period, the more pronounced the differences in health belief scores became. This suggests that telemedicine-based health education is more effective in improving the health beliefs of older participants with T2D compared to traditional health education [36]. Health education makes participants aware of the complications, severity, and harms associated with their condition, as well as the benefits of adopting a healthy lifestyle. This encourages them to take the initiative in managing their health and deepens their understanding of diabetes. In addition, the feedback and reminders provided by telemedicine play a positive role in enhancing participants’ health beliefs [37], which strengthens their health motivation and helps them establish a correct and comprehensive set of health beliefs. The results of this study show that health education based on the HBM within the context of telemedicine has a positive impact, increasing patient self-efficacy, reducing perceived barriers in the intervention group, and facilitating an increase in patient health behaviors.

We also found that the intervention improved self-efficacy in older adults with T2D, similar to the results of other studies [38]. Given that increased health beliefs are associated with higher diabetes self-efficacy [20], participants in the intervention group experienced higher self-efficacy as their level of health beliefs increased. Elevated levels of self-efficacy are often linked with decreased barriers to engaging in health behaviors, which in turn leads to increased motivation to engage in desired activities [39].

### Limitations

First, while studies have shown the short-term impact of telemedicine on glycemic management in older adults with T2D, the long-term validity of these effects remains unassessed. Second, the participants who chose to join this study likely demonstrated some level of readiness for digital health literacy change. Therefore, strategies have yet to be developed for those uninterested individuals who did not participate in the study. Finally, the study relied on structured questionnaires for participants to self-assess their self-efficacy, health beliefs, and diabetes self-management skills, which may have introduced recall bias into the results.

### Conclusions

The findings of this study suggest that health education and telemedicine interventions based on the HBM can improve HbA_1c_ levels and other related outcomes in older adults with T2D. The telemedicine system provided timely access to the health status of older participants with T2D, facilitated communication and feedback between doctors and participants, and enabled the inclusion of health care provider counseling to enhance patient motivation. This, in turn, improved the effectiveness of medical care, leading to better glycemic management in the intervention group. Future research should further evaluate the long-term effects of this approach compared to routine community diabetes care. In addition, it should focus on providing better support for lifestyle changes through enhanced interventions and counseling to improve patient motivation and confidence, ultimately offering more comprehensive diabetes management for participants.

## Supplementary material

10.2196/60227 Multimedia Appendix 1 Mean andCIs of glycosylated hemoglobin, summary of diabetes self-care activities measure, self-efficacy for diabetes and Health Belief Model scores for the 2 groups in the study.

10.2196/60227 Checklist 1 CONSORT (Consolidated Standards of Reporting Trials) checklist.

## Abbreviations

CONSORT: Consolidated Standards of Reporting Trials
HbA_1c_: glycosylated hemoglobin
HBM: Health Belief Model
SDSCA: summary of diabetes self-care activities measure
SED: self-efficacy for diabetes
T2D: type 2 diabetes
