# Supplementary material for: Community-Based Intelligent Blood Glucose Management for Older Adults With Type 2 Diabetes Based on the Health Belief Model: Randomized Controlled Trial
Source: JMIR Mhealth Uhealth. 2025 Jun 19;13:e60227. doi: 10.2196/60227 (PMC12199844; doi:10.2196/60227)
Supplement: Multimedia Appendix 1 [file mhealth-v13-e60227-s001.docx]

Online-Only Supplemental Material

Figure S1 Mean and confidence intervals of glycated hemoglobin levels in both groups

Figure S2 Mean and confidence intervals of SDSCA, SED and HBM scores for the two groups in the study


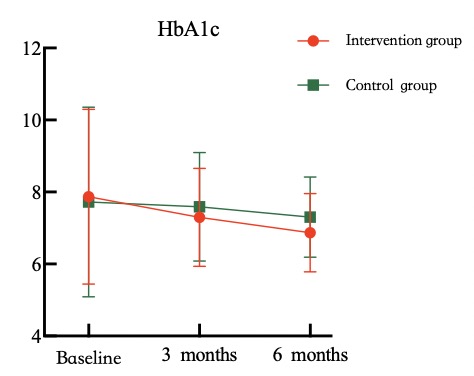


Figure S1 Mean and confidence intervals of glycated hemoglobin levels in both groups


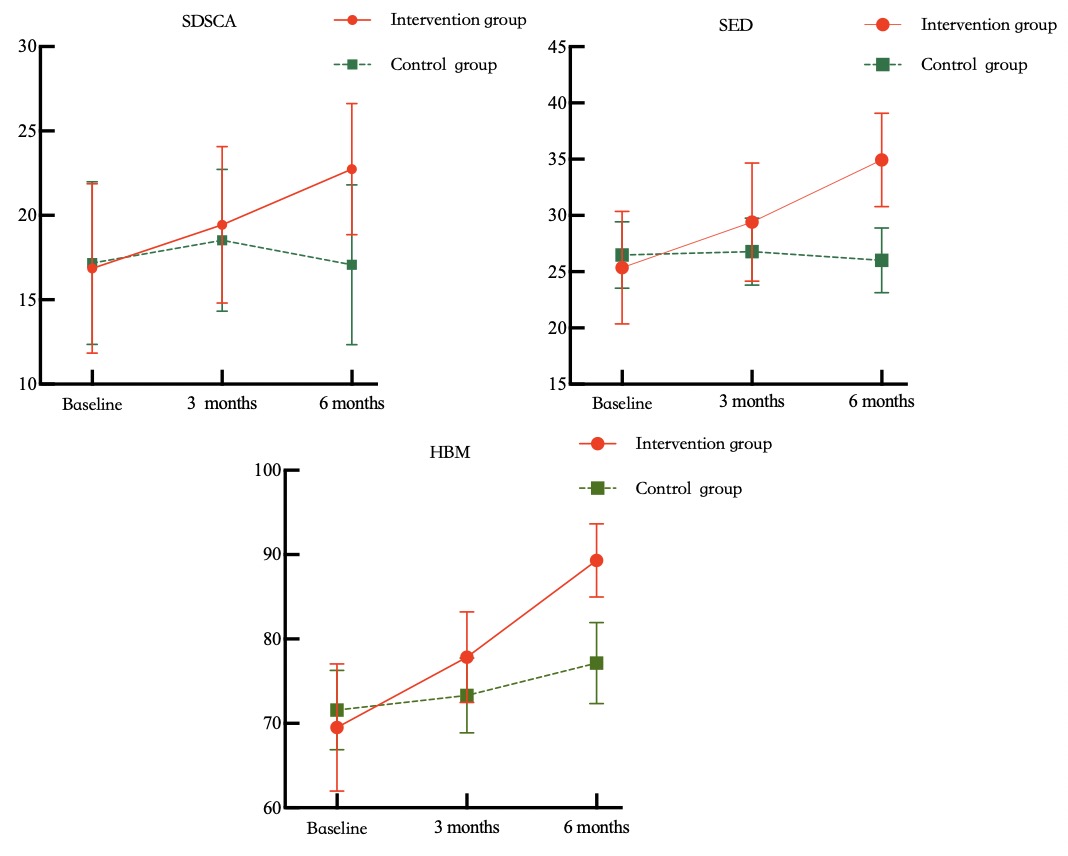


Figure S2 Mean and confidence intervals of SDSCA, SED and HBM scores for the two groups in the study
